# Assessing the Usability of a Novel Toolkit for Creating Visual Key Information Pages for Informed Consent for Research: Mixed Methods Usability Study

**DOI:** 10.2196/76740

**Published:** 2025-11-20

**Authors:** Eliana Charlotte Goldstein, Zoe Troubh, Krista E Cooksey, Molly Volkmar, Victor Catalan Gallegos, Kimberly A Kaphingst, Clara N Lee, Ashley J Housten, Jessica Mozersky, Mary C Politi

**Affiliations:** 1 School of Public Health, Washington University in St. Louis, 4300 Duncan Ave, Room 2110, St. Louis, MO, 63110, United States, +1 314-935-3802; 2 Washington University in St. Louis, St. Louis, MO, United States; 3 Huntsman Cancer Institute, University of Utah, Salt Lake City, UT, United States; 4 Department of Surgery, Division of Plastic and Reconstructive Surgery, School of Medicine, University of North Carolina at Chapel Hill, Chapel Hill, NC, United States; 5 Department of Communication, University of Utah, Salt Lake City, UT, United States; 6 Department of Surgery, Division of Public Health Sciences, School of Medicine, Washington University in St. Louis, St. Louis, MO, United States; 7 Department of Medicine, Division of General Medicine and Geriatrics, School of Medicine, Washington University in St. Louis, St. Louis, MO, United States

**Keywords:** informed consent, health literacy, key information, usability testing, think-aloud protocol, qualitative mixed-methods

## Abstract

**Background:**

Key information pages for informed consent require a concise summary of information to improve participant understanding but have not widely incorporated health literacy best practices.

**Objective:**

We previously developed a visual key information template to improve informed consent. In this study, we conducted usability testing of this customizable one-page key information template.

**Methods:**

We used the Designing for Accelerated Translation framework to plan for actionable, efficient usability testing. Participants (N=15) were asked to spend about 20 minutes using the visual key information template and engaging in a think-aloud protocol. They then responded to qualitative debrief questions about the template and validated measures of acceptability, appropriateness, and feasibility. Interviews were recorded, transcribed, and analyzed with a usability-focused codebook and thematic analysis.

**Results:**

The toolkit was positively received. Common usability challenges included interpreting instructions, condensing consent content, replacing and resizing icons, and fitting information into template boxes. Participants had positive experiences with toolkit elements, particularly with the icon library, and generally felt the toolkit was easy to use and encouraged simplification of information. Some participants noted not fully reviewing instructions before the study and discussed specific technical abilities as potential limitations of widespread use. We documented suggestions and made changes to the toolkit in response to feedback received.

**Conclusions:**

Overall, participants considered the toolkit to be appropriate, acceptable, and feasible. Additional implementation outcomes are being collected in a multisite stepped-wedge randomized trial. Further research may investigate changes to format and software that balances functionality with ease of use.

## Introduction

### Background

Informed consent documents for research studies aim to introduce research participants to important topics including research design, risks and benefits, data privacy, and participant rights. Research and regulatory oversight teams often struggle to clearly present these complex details, which results in consent forms that are lengthy, repetitive, and difficult to understand [1-5]. The introduction of the key information (KI) page in the 2018 Federal Common Rule sought to address these challenges by requiring a “concise and focused summary” of information most important to potential participants who are deciding whether to join a study [6]. Although evidence-based recommendations for how best to use KI pages are limited to date, the guidance that does exist encourages flexibility to adapt information to the audience [7,8]. Existing research has explored the use of visuals and health literacy best practices in informed consent broadly [9-13]; however, to our knowledge, there is no available, evidence-based guidance, or toolset for integrating these elements into the KI page specifically.

### Prior Work

In previous research, our team developed and tested options for a one-page KI template incorporating both visual elements and health literacy best practices [14,15]. The goal of this template is to support existing consent best practices with an appealing, clear, and actionable first page of research consent forms. We compared four potential templates for visually enhanced key information pages designed by either our research team or a nonprofit communications organization that specializes in disseminating health information in an accessible way [14]. The options were qualitatively reviewed by 40 users (principal investigators, research staff, institutional review board (IRB) members and staff, and community members) and iteratively updated in response to their feedback. The key elements of the final template preferred by users included organizational boxes with contrasting headers, icons, color, bulleted text, and ample white space, with accurate, accessible, and actionable information, consistent with health literacy best practices [16]. In parallel, we conducted pilot testing of templates leveraging similar elements in four ongoing studies [15]. In pilot tests, participants reported high knowledge of study details, high approval of the visuals, and low conflict about joining studies. These pilots also served as an opportunity to identify and address important implementation considerations and refinements of the template to ensure adaptability for varying study types and subjects.

After selecting and refining our approach, we developed a customizable toolkit in Microsoft PowerPoint [14,17]. The toolkit includes an editable version of the template, instructional documents and videos, an icon library, and examples to empower study teams to use the proposed template as a replacement for text-only key information pages in informed consent documents.

### Study Objectives

Our first phase of research collected feedback on already-created key information templates but did not ascertain if the resources in the resulting toolkit would be usable for research teams interested in creating these pages for their own studies. This paper describes mixed-methods usability testing of the toolkit elements and template as part of our rapid dissemination and implementation approach to ensure the toolkit would be acceptable, appropriate, and feasible for use by interested research teams.

## Methods

### Frameworks

Our approach to the design, implementation, and dissemination of this intervention was guided by the Designing for Accelerated Translation (DART) framework [18]. The DART framework outlines the connections between three elements key to quickly putting innovations into practice: (1) forming meaningful partnerships; (2) designing for dissemination and implementation; and (3) engaging with a learning system to iteratively adapt and refine materials. The protocol for this study responds to these elements (Table 1), with the goal of rapidly assessing and responding to common usability issues with the proposed template and toolkit.

**Table 1.** Usability study protocol elements responding to the Designing for Accelerated Translation (DART) Framework [18].

DART element	Protocol element
Form meaningful partnerships	Engage with likely end users as participants (principal investigators, research coordinators, or research support staff) Collaborate with the institutional review board to ensure the visual KI pages could be integrated routinely
Designing for dissemination and implementation	Select commonly available software for the toolkit Provide proposed resources to support users Update resources in response to feedback Monitor interviews for thematic saturation
Learning systems for rapid adaptation	Encourage participants to test template using their specific consent and study protocol Provide poststudy support to participants interested in implementing visual key information into their study Track adaptations using a standard framework

We based our coding framework on previous studies of usability of computer-based job tools, which organized usability challenges within four domains: navigation, content, functionality, and layout [19,20]. Additional description of codebook development is included below. To track suggested changes beyond the scope of the codebook, we used the Framework for Reporting Adaptations and Modifications to Evidence-Based Interventions (FRAME) [21].

### Participant Eligibility and Recruitment

We invited principal investigators and research staff at three large research universities with affiliated medical schools (one in the Midwest, one in the South, and one in the Mountain West) to complete usability testing via email. Potential participants were identified from previously compiled lists of potential participants developed based on peer recommendations of site lead investigators (KK, CL, MP) and targeted searches for people in relevant roles with publicly available email addresses, as well as via snowball sampling and word-of-mouth referral [22]. Eligible participants included (1) principal investigators, (2) research team members (eg, coordinators, project managers), or (3) research support staff with a focus on protocol development or recruitment. Based on standard sample sizes for usability testing [23], we planned to recruit 10‐15 participants in an initial round of recruitment, with an approximately even split across the three categories of professional roles. If new usability considerations were identified in the last 5 interviews, the research team planned to recruit additional participants.

### Data Collection

We designed a think-aloud protocol for usability testing based on examples from the literature [19,20,24]. We sent study materials (the visual KI toolkit described above, a brief summary document, and a 5 min introductory video) to participants in advance of scheduled interviews for their review and preparation. Participants spent approximately 20 minutes filling out the visual KI template based on an existing consent form (ie, their own or an example provided by the study team, per participant preference) before the research team asked them to stop. Participants were asked to screenshare using video conference software as they completed the task. Interviewers prompted participants with a predetermined list of questions if they were silent for more than 20 seconds (Multimedia Appendix 1). To minimize participant burden, participants were instructed to complete as much of the template as they were able during the allotted time; there was no necessary minimum completion. After testing, interviewers collected responses to debrief questions and validated measures of acceptability, appropriateness, and feasibility each rated on a 5-point scale (strongly disagree=1 to strongly agree=5) [1,25]

### Data Analysis

Interviews were recorded, transcribed, and divided into observational and debrief portions for coding [26]. A usability-focused codebook was developed by three research team members (EG, KC, MP), based on anticipated challenges and challenges that emerged from the first five participant interviews and refined as needed throughout the coding process. The observational portions of the interviews were each coded to 21 total codes by two coders (EG, ZT). Coding was compared to identify discrepancies (ie, one coder selected a code while the other did not). Coders then met to review the discrepancies and consulted a third coder (KC) to reach consensus for each transcript. The average percent agreement across all transcripts was 85% prior to consensus meetings. A challenge was considered frequent if at least eight (>50%) of participants experienced it one or more times in their interview.

Several research team members (EG, MV, VC, KC) developed additional codes to analyze the broader scope of the debrief portion of the interviews, using an inductive methodology described by Naeem et al [27]. Debrief portions were coded collaboratively by the two coders (EG, ZT) with consultation from the third coder (KC). Acceptability, appropriateness, and feasibility scores were calculated based on standard methodology for the validated measures [25].

### Ethical Considerations

The Washington University in St. Louis Institutional Review Board approved this study as exempt research (IRB #202405126). The research team reviewed consent information with participants and confirmed their interest in joining the study and being recorded to observe study procedures, prior to beginning the usability interview. Written transcripts were deidentified prior to depositing in a data repository. Data are available in the repository via restricted access with a study protocol, ethics approval, and rationale for access [26]. Participants received a US $50 gift card after completing all study procedures.

## Results

### Participants

Fifty-one eligible potential participants were invited to participate. Of those, 16 agreed to participate, 2 declined, and 33 were unable to be reached. A total of 15 participants completed 14 total interviews (Table 2). In one interview, 2 participants working on the same research project requested to be interviewed simultaneously, with 1 participant controlling the visual KI template while the other offered suggestions and commentary.

**Table 2.** Usability interview participant demographics (N=15).

Demographic characteristics	Participants (N=15)
Age (years)	
Mean (SD)	44.2 (12.57)
Range	27‐76
Gender, n (%)	
Woman	10 (66.7)
Man	4 (26.7)
Another response	1 (6.7)
Ethnicity, n (%)	
Latino/a/Latine or Hispanic	1 (6.7)
Not Latino/a/Latine or Hispanic	14 (93.3)
Race, n (%)	
White	14 (93.3)
Asian	1 (6.7)
Institutional role, n (%)	
Principal Investigator (PI)	6 (40.0)
Research Support Team Member (eg, clinical trial oversight, recruitment enhancement)	3 (20.0)
Research Team Member (eg, coordinators, managers)	6 (40.0)

### Observational Period Findings

We assessed the number of participants who reported each identified usability challenge (Table 3). Five frequently observed challenges were noted; 2 were related to content in the toolkit: participants had difficulty interpreting instructions and participants had difficulty condensing content from the full consent document into the visual KI template. Two challenges were related to functionality: participants had technical issues updating information in boxes and participants had trouble replacing or resizing icons. One was related to layout: participants had challenges fitting study information into the boxes provided. There were few navigation challenges other than navigating between pages, which occurred among 4 participants.

**Table 3.** Number of participants reporting usability challenges and adaptations made to address challenges (N=15).

Usability challenge	Participants (n, %)	Adaptations to address challenge
Content		
Participant had difficulty interpreting instructions.	8 (53.5)	Clarified instructions; developed demonstration video resource
Participant had difficulty condensing content from full consent into bullets.	8 (53.3)	Developed demonstration video resource
Participant had difficulty splitting content across boxes.	7 (46.7)	Developed demonstration video resource
Icon not available for participant’s desired image.	4 (26.7)	Maintaining list of suggested icons
Participant expressed desire to include information beyond the recommended scope of visual KI.	3 (20.0)	N/A^a^
Participant indicated specific information was unavailable/missing.	2 (13.3)	Clarified instructions; developed demonstration video resource
Functionality		
Participant had trouble replacing or resizing icons.	10 (66.7)	Reviewed formatting to ensure appropriate layering of elements; developed demonstration video resource
Participant had technical issues updating information in boxes.	9 (60.0)	Reviewed formatting to ensure appropriate layering of elements; developed demonstration video resource
Participant had other technical issues.	3 (20.0)	N/A
Participant discussed a goal/functionality that cannot be achieved in PowerPoint.	2 (13.3)	Maintaining list of suggested future functionalities
Participant had difficulty manipulating size of boxes or box headers.	1 (6.7)	Developed demonstration video resource
Layout		
Participant said they could not fit desired study information in the boxes provided.	8 (53.3)	Developed demonstration video resource
Participant expressed that the text is too small.	4 (26.7)	Developed template version with larger font size
Participant had trouble creating text wrap.	3 (20.0)	Reviewed formatting to ensure appropriate layering of elements; developed demonstration video resource
Participant had trouble reading side instructions.	1 (6.7)	N/A
Navigation		
Participant had difficulty moving from page to page in toolkit.	4 (26.7)	N/A
Participant unintentionally moved items around.	3 (20.0)	Reviewed formatting to ensure appropriate layering and grouping of elements
Participant unintentionally changed format.	3 (20.0)	Reviewed formatting to ensure appropriate layering and grouping of elements
Participant had difficulty finding desired icon.	2 (13.3)	N/A
Participant initially had difficulty determining which template to use (6 vs 8 box).	1 (6.7)	Developed demonstration video resource
Participant expressed unfamiliarity with PowerPoint.	1 (6.7)	N/A

a Not applicable.

### Debrief Period Findings

In the debrief portion of the interviews, participants indicated that the toolkit was overall easy to use. They noted that the templated boxes made the task straightforward and encouraged them to simplify some information:

Having these headings at the top of each [box] helps to also organize the information just mentally before you’re getting started. You can already start thinking about what goes where and what might make sense to a potential participant or patient who’s reviewing this.

[P10, research support team member]

I think that having a toolkit like this forces you to write things fairly simply and directs you exactly what to do.

[P14, principal investigator]

Some participants referred to their experience as “intuitive” (P06, research team member; P14, principal investigator). Others highlighted that the “inherent flexibility” (P08, principal investigator) of the toolkit met their needs and speculated it would meet others’. However, some noted that the template by itself did not necessarily solve all challenges with the content of informed consent forms, such as a high volume of desired content or a tendency toward jargon:

It’s hard, especially the goal of the study, if there’s a lot involved, it was hard for me to figure out what to put there.

[P12, research team member]

I like the idea, but I think what…everybody has to do…is that you need to check the level of language. Because all of us are wrong all the time.

[P01, principal investigator]

Participants also responded positively to the resources in the toolkit, particularly the icon library.

I love that [the icon library] is there…it’s easy to get lost in a rabbit hole of looking for the right icon and then trying to figure out any copyright privileges...knowing that there’s a library of things that I can select from was nice.

[P07, principal investigator]

Not all participants used the resources provided. For example, multiple participants noted that they had not read through the instructions. Some felt the instructions were too long but others appreciated inclusion of background and technical details:

These first two pages being so dense…I was like ‘Oh, I just want to know what I need to do right now.

[P06, research team member]

I’ve never really seen anything really like it other than just templated fliers…but [with those] you’re sort of just on your own. I think it’s important to…have that instruction and…an educational piece.

[P11, research support team member]

Participants noted that users’ technical ability or experience could limit feasibility of use. One participant was also concerned about the possibility of users inadvertently creating unappealing materials with the template:

People who know how to do it, right, they’re going to do it. If this is required to be used, they’re going to hate it. People who don’t—if they don’t have that skill in design principles anyway, they’re going to [mess] it up.

[P09, research support team member]

### Suggestions and Adaptations

Suggestions that arose throughout the interviews were documented and iteratively considered for implementation. In addition to the changes made to address usability issues (Table 3), several revisions were made based on specific recommendations from participants:

- Updated the “default” set of icons on template pages to ensure they are as broadly applicable as possible
- Moved the icons to the bottom right of boxes to minimize issues with text wrapping
- Added further guidance about options for customization
- Clarified best practices for using icons in payment box to minimize sources of confusion

Large-scale changes to the format of the template (eg, adding new visual elements like arrows between boxes, migrating toolkit to a platform that would allow dropdown selection of icons) were beyond the scope of this usability testing; however, these suggestions were documented using the FRAME for potential future work. We are also maintaining a list of proposed additions to the icons for future iterations.

### Toolkit Acceptability, Appropriateness, and Feasibility

All 15 participants completed all acceptability, appropriateness, and feasibility measures. The toolkit was generally positively received, with most participants rating it highly across 5-point acceptability, appropriateness, and feasibility scales: mean acceptability score=4.28 (range: 1.50‐5.00; SD 0.87); mean appropriateness score=4.32 (range: 3.00‐5.00; SD 0.57); mean feasibility score=4.35 (range: 2.75‐5.00; SD 0.63) [25].

## Discussion

### Principal Results

These usability interviews affirmed previous findings that the proposed visual KI template is an acceptable tool for researchers conducting human subjects research [14]. Participants generally found the toolkit for key information both straightforward and flexible to meet their study’s needs. Participants in our prior research suggested that the boxes in the toolkit would be useful for participants’ comprehension and experience [14]; in this work, we found they are also helpful for researchers thinking through how to write and summarize key consent information. However, it is important to ensure that the flexibility of the template is clear and that instructions are modular so that users can customize the content to fit study-related needs and preferences while meeting overall key information best practices. We refined instructions and created a demonstration video resource to ensure users have access to clear information about when and how to customize major elements of the template.

Although the toolkit addresses many of the formatting issues that can make informed consent forms unappealing or intimidating, participants continued to report that selecting appropriate content for key information remains a challenge (ie, what information is appropriate to include, how to “translate” from jargon-filled protocols to plain language). The template helps to suggest some overall content categories but does not provide a complete solution to helping individuals summarize important study-related material. This research provided a first step focusing on usability challenges specific to a previously developed informed consent toolkit; future research could explore how to select the most appropriate content for the toolkit within the box headings.

Our selection of Microsoft PowerPoint as the platform for this toolkit has meant that we are unable to address some of participants’ specific functionality desires such as including drop-down menus for icon selection. However, we chose to use this program given its broad accessibility in the United States and users’ overall comfort with it. Many participants informed us in our previous studies, that introducing a new software would create steep learning curves for those less familiar with technology [14]. Future work could explore the best software to balance ease of use with functionality.

This toolkit goes beyond previous evidence-building and training modules in the area of informed consent improvement [11,12] by offering an adaptable template that is available for use by interested research teams [17]. An editable toolkit like this is substantially more actionable than a video or in-person training. Most participants in this study indicated that the toolkit provided immediate and obvious value to their consent development process, where noninteractive trainings or standalone design examples do not prompt hands-on learning with a usable output. We integrated trainings and design examples as optional supplemental resources for those who want additional guidance rather than the primary elements of the intervention. Research teams developing interventions that require end users to create highly tailored content should consider similarly editable tools with integrated instructions to promote flexible, “learn as you work” approaches for busy professionals.

The usability testing described above was a critical step in ensuring that any obvious barriers to using the toolkit were addressed prior to wider dissemination. The think-aloud protocol was an efficient, effective, and flexible approach to refining the toolkit that others working to create interactive job aids or templates may consider incorporating into their development process. As suggested in the DART framework, engaging end users in qualitative testing has also helped us identify “power users” within research and research support teams who act as early adopters of the toolkit and advocate for its use among their peers [18].

### Limitations

These usability interviews were conducted with a limited number of participants working at similarly-resourced academic institutions in the United States; results might not generalize to participants in other institutions. The small sample size included many people with an existing interest in improving informed consent, which may limit our findings of acceptability, appropriateness, and feasibility. In addition, participants may have worked through challenges that, if they were not being observed, may have caused them to give up while using the toolkit on their own. However, we did not note any participants expressing significant frustration during the interview period. About half of participants worked on the task based on their own study protocols or consents (n=8 participants with 7 unique protocols), but many used a sample consent for a previous decision support intervention project. This could have limited the number of study type-specific challenges encountered, particularly because only one protocol included a medication intervention.

### Conclusion

Participants generally found the proposed KI toolkit appropriate, acceptable, and feasible. Some usability issues were addressed with enhanced instructional resources, and other suggestions related to the toolkit content were considered and incorporated. Designing our study with reference to elements from the DART framework helped ensure that insights could be implemented quickly in routine use. These updates are contributing to our ongoing testing and dissemination work, which includes a multisite stepped-wedge randomized trial to build evidence for implementation and effectiveness outcomes; collaborations with IRBs to create approved, site-specific workflows for submission and review; and live and asynchronous training and support opportunities for researchers and research staff. Future research could explore guidance for selecting the right content for KI pages and technology opportunities (eg, some have suggested exploring health-literacy-informed prompts for generative artificial intelligence to simplify or shorten consent content for input into the template) that provide more tailored support for users developing visual KI pages.

## Supplementary material

10.2196/76740 Multimedia Appendix 1 Think-aloud prompts used with participants after approximately 20 seconds of silence.

## Abbreviations

DART: Designing for Accelerated Translation
FRAME: Framework for Reporting Adaptations and Modifications to Evidence-Based Interventions
IRB: institutional review board
KI: key information
