# Supplementary material for: Assessing the Usability of a Novel Toolkit for Creating Visual Key Information Pages for Informed Consent for Research: Mixed Methods Usability Study
Source: JMIR Form Res. 2025 Nov 20;9:e76740. doi: 10.2196/76740 (PMC12634007; doi:10.2196/76740)
Supplement: Multimedia Appendix 1 [file formative-v9-e76740-s001.docx]

**Appendix A. Prompt Questions for Think Aloud Protocol**

- General
  - If they stop clicking/writing/etc. for more than a few seconds
    - What are you thinking about right now?
    - Is there something you’re stuck on? [if yes: What is it?]
  - If they seem to be struggling with a task—expressing nonspecific frustration, trying to do same thing repeatedly, etc.
    - Is there anything you’re finding difficult or frustrating right now?
    - What is making this task difficult?
    - Is there information that would be helpful about how to do this task?
- Boxes
  - When they are selecting 6 v 8 box template
    - How did you decide which template to use?
  - If they change box headers
    - Why did you decide to change the box header?
- Content
  - Ask after they’ve begun adding some text
    - How are you deciding what information to include?
    - What would make your decisions easier?
    - Are you rewriting anything from the original protocol? [if yes: Why? How are you thinking through your edits?]
- Icons
  - While choosing icons
    - Why did you pick that icon?
    - [if they are struggling] What information would make your decisions easier?
